# The Treatment of Surgical Shock by Saline Infusions*Read before the El Paso County Medical Society, El Paso, Texas, March 11, 1899.

**Published:** 1899-04

**Authors:** J. Shelton Horseley

**Affiliations:** El Paso, Texas


### TEXAS MEDICAL JOURNAL.

ESTABLISHED JULY, 1885.

PUBLISHED MONTHLY.—SUBSCRIPTION $1.00 A YEAR.

VOL. XIV. AUSTIN, APRIL, 1899. No. 10.

### Original Contributions.

For the Texas Medical Journal.

### The Treatment of Surgical Shock by Saline Infusions.*

BY J. SHELTON HORSELEY, M. D., EL PASO, TEXAS.

In days gone by when malaria, liver complaint and worms covered a multitude of the physician's sins, shock did the same thing for the surgeon. The last decade, however, has done much to clear up this *terra incognita* in the province of both the physician and the surgeon, so that with this advance shock is being viewed in a far more intelligible light and the raids of the bugbear, though not entirely done away with, have been greatly lessened.

When it was learned that surgical shock was due to anemia of the brain, a great step had been taken in the right direction, but only when the etiology of cerebral anemia became fully understood could a rational treatment be carried out. As soon as it was discovered that a lessened amount of blood to the brain more frequently resulted from collateral hyperemia than from a direct loss of the fluid itself, and that an animal might, so to speak, bleed to death in its own blood vessels, it became evident that the gravity of an operation would be proportionate to the violence done the nervous system, directly or reflexly, as well as to the amount of blood lost. It followed that there were two indications for treating surgical shock, namely, to cause contraction of the arterioles and so diminish the space to be filled by a given amount of blood; or, to cause increase

*Read before the El Paso County Medical Society, El Paso, Texas, March 11, 1899.

in the volume of blood to fill the given space. The first indication is uncertain in its accomplishment, which is dependent upon the profundity of the shock and the idiosyncrasies of the patient. The usefulness of drugs in cases of grave shock is, to say the least, dubious. If the cerebral centers are profoundly depressed, they often fail to respond to the action of any drug stimulation.

To fill the second indication, i. e., to increase the volume of blood to fill the given space, is by far the more rational and certain method of treatment, and to this end the modern treatment of shock tends.

When marked shock occurs, either with or without hemorrhage, it is a waste of most valuable time to rely solely on hypodermic medication when it it is a demonstrated mechanical fact that lowering of the head and intravenous injections of hot, sterile salt solution will bring the needed blood to the anemic brain.

And too frequently the infusion of salt solution is not employed as a prophylactic measure, when it is clearly indicated as such. Patients who have already lost blood either as the result of an accidental injury or of some pathological process, as fibroids of the uterus, should usually receive a saline infusion before any operative procedure is attempted.

Greig Smith has most aptly said: "The prime object in surgery is not to perform a scientific and technically complete operation, but to save life." Instead then of rushing through an operation with a patient profoundly shocked, if the surgeon would immediately infuse with salt solution he would find eventually there would be fewer deaths in his mortality rate.

As to the preference between cellular infusion and intra-venous infusion, the latter seems more desirable. In any case in which a saline infusion is indicated, the symptoms must be more or less urgent. If the circulation is feeble the intra-cellular infusion will necessarily be slowly absorbed. The salt solution cannot be injected at a high temperature, and even then will be likely to lose some of its heat before it reaches the heart, whereas the application of heat directly to the cardiac walls by means of the hot saline solution introduced in the veins is, of itself, a strong stimulant to the heart. If placing a hot towel over the cardiac area of the chest wall is a stimulant to the heart, surely heat directly applied to this organ must be more beneficial. An intra-venous saline infusion should be of a temperature of about 110° to 115° F., or as hot as the hand can bear. Dr. Dawbarn and Dr. Kemp, of New York City, have proven experimentally that an intra-venous injection of normal salt solution at a temperature of 120° F. can be used without deleterious effect, but that above this temperature the blood corpuscles and the tunica intima of the veins are injured; and that this solution between 110° and 120° F. is more efficient in overcoming shock than one containing less heat.

Dr. Howard A. Kelly is one of the few prominent operators who prefers the intra-cellular to the intra-venous method. He has not studied the subject experimentally. The vast majority of prominent surgeons of the Eastern cities use only the intra-venous method for the reasons already mentioned. Dr. Horace Tracy Hanks, of New York, in an article in the *American Gynecological and Obstetrical Journal* for September, 1898, says he has tested the intravenous method in the treatment of shock for the past three years, and has had no accident following its use. He infuses at a temperature of 115° F. from one pint to three quarts of the solution, and uses this method for loss of blood from any cause; for severe traumatism; for the early stage of sepsis after an operation; for suppression of the urine, and for obstruction of the bowels from paralysis after laparotomy.

The three chief objections urged against intra-venous infusions are: (1) The entrance of air into the veins; (2), the difficulty of getting the solution perfectly clear without floating particles; and (3), troublesome apparatus and technique. The first objection is the one most often brought forward, but if the canula is introduced into the vein while the solution is flowing from it and taken away before the solution is quite used up, it will be impossible for air to gain entrance. Besides, even if it does, the matter is not of such great moment as is generally supposed. Several minims of air can be introduced into the veins of a rabbit with a hypodermic syringe without untoward effects. Of course larger quantities would prove fatal, but a small amount generally becomes lodged in the sinus behind one of the valves of the heart, and is gradually dissolved in the blood current. It is a physiological fact that normal blood serum contains a considerable amount of nitrogen and some oxygen dissolved in it. As to the second danger, the solution can be readily passed through a filter composed of sterile gauze and cotton, and any small floating specks found after this filtration are harmless. The third objection will be answered by a description of the technique later on.

In the intra-cellular method of infusion normal salt solution should be used, which is a six-tenths of one per cent. solution of sodium chloride in water. This should be boiled in a porcelain or agate-ware vessel, and allowed to cool. It may be brought to the proper temperature for injection (100° to 102° F.) by adding a hotter solution. A fountain syringe, or better a glass percolator, or glass irrigating apparatus, should be connected by a rubber tube six feet long with an aspirating needle. The needle must be inserted under the mammary gland, or under the skin of the abdomen. An elevation of about three or four feet must be maintained, and after the skin has become quite tense the needle must be withdrawn and inserted elsewhere.

The apparatus necessary for intra-venous infusions is of the simplest. The irrigator should be of glass, though a fountain syringe will do. The bottom knocked out of a half-gallon bottle, the bottle inverted and a cork perforated by a glass tube placed in the neck is entirely satisfactory. The most essential thing is that the apparatus be sterile. A fountain syringe, rubber tubes and small glass tubes should be boiled; large glass receptacles should be sterilized by a solution of bichloride of mercury (1 to 1000) and finally washed with plain sterile water. The best thing for entering the vein is an ordinary straight glass medicine dropper. The solution should be normal salt solution, as anything else, even plain sterile water, has been shown experimentally to dissolve the blood corpuscles and would produce a fatal result. The solution should be sterilized by being first boiled in a porcelain or agate-ware vessel and cooled. Hot solution may be added to produce a temperature of about 117° F., and this then passed through a filter of sterile gauze and cotton. The elevation must be about three feet from the level of the vein.

The operation is begun by making ordinary preparations for carrying out an aseptic procedure, the arm corded just enough to arrest the venous circulation, and an incision of about one inch made over some prominent vein on the front of the elbow, usually the median-basilic. The skin over this vein is caught up with forceps and incised in the direction of the axis of the vein, then by cutting a thin layer of fascia the vessel is fully exposed. It should be completely grasped at the lower end of the incision by an artery forceps, and the wall of the vein just on the proximal side of the hemostat caught with tissue forceps, opened with scissors, and the medicine dropper inserted while the solution is running. The cord may then be removed from the arm. From one pint to three quarts of normal salt solution should be thus infused, the quantity used depending upon the amount of blood lost and the profundity of the shock.

